# Reframing Patient Experience Approaches and Methods to Achieve Patient-Centeredness in Healthcare: Scoping Review

**DOI:** 10.3390/ijerph19159163

**Published:** 2022-07-27

**Authors:** Eun-Jeong Kim, Inn-Chul Nam, Yoo-Ri Koo

**Affiliations:** 1 Department of Otorhinolaryngology-Head and Neck Surgery, The Catholic Medical Center, The Catholic University of Korea, Seoul 06591, Korea; dodam.design.research@gmail.com; 2 Department of Otorhinolaryngology-Head and Neck Surgery, Incheon St. Mary’s Hospital, The Catholic University of Korea, Seoul 21431, Korea; 3 Department of Service Design, Graduate School of Industrial Arts, Hongik University, Seoul 04066, Korea

**Keywords:** patient experience, patient-centered care, design thinking, holistic approach, creative problem-solving, multidisciplinary perspective

## Abstract

(1) There has been growing attention among healthcare researchers on new and innovative methodologies for improving patient experience. This study reviewed the approaches and methods used in current patient experience research by applying the perspective of design thinking to discuss practical methodologies for a patient-centered approach and creative problem-solving. (2) A scoping review was performed to identify research trends in healthcare. A four-stage design thinking process (“Discover”, “Define”, “Develop”, and “Deliver”) and five themes (“User focus”, “Problem-framing”, “Visualization”, “Experimentation”, and “Diversity”), characterizing the concept, were used for the analysis framework. (3) After reviewing 67 studies, the current studies show that the iterative process of divergent and convergent thinking is lacking, which is a core concept of design thinking, and it is necessary to employ an integrative methodology to actively apply collaborative, multidisciplinary, and creative attributes for a specific and tangible solution. (4) For creative problem-solving to improve patient experience, we should explore the possibilities of various solutions by an iterative process of divergent and convergent thinking. A concrete and visualized solution should be sought through active user interactions from various fields. For this, a specific methodology that allows users to collaborate by applying the integrative viewpoint of design thinking should be introduced.

## 1. Introduction

### 1.1. Patient Experience (PE) Approaches and Design Thinking (DT) as a Creative Problem-Solving Method

Recently, PE for quality improvement (QI) has increasingly evoked interest among researchers [1]. To achieve high-quality care in medical services, patient-centeredness should be considered a key attribute of healthcare [2,3]. Patient-centered care is defined as (1) being responsive to patients’ needs, values, and preferences; and (2) involving patients in decision-making [3,4,5]. Accordingly, efforts to improve PE through patient engagement in healthcare are intensified. PE involves several stakeholders, such as patients, caregivers, medical staff, and administrators, dealing with complex and diverse problem situations. However, the existing traditional healthcare method for patient engagement has limited the patient’s role in the problem-solving process by putting healthcare experts and researchers in the center of PE improvement [1], which resulted in the patient’s passive attitude toward their quality of care.

As a new way to engage patients, participatory design, experience-based co-design (EBCD), and co-design were introduced by employing service design methods such as service blueprints, user journeys, and stakeholder maps. These methodologies are relatively unfamiliar design approaches derived from the concept of DT and were originally meant as a holistic and integrative process and methods for problem-solving by using research, prototyping (visualized evidence), and co-creating values for relevant stakeholders [1,6,7]. DT is also an approach that iteratively repeats the process of diverging and converging ideas by engaging all relevant users in a human-centered and collaborative manner [6,8]. The converging process corresponds to the concept of narrowing down choices to find a solution to a specific problem. In contrast, the diverging process considers the problem space from a new perspective and reviews the situation in various creative ways that were not previously thought of. Since DT holistically and iteratively deals with the overall process for problem-solving, from problem recognition and definition, from a new perspective to problem identification and ideation, and for solutions to a concrete presentation of visualized results, it can serve as a useful viewpoint and methodology in solving complex and diverse problems from a patient perspective in healthcare. Stakeholders’ views are actively reflected in the solution by conducting collaborative work among users to deeply understand interrelated human activities.

Therefore, it provides a holistic and integrative view of exploring the complex problems of healthcare [6,9,10,11]. This approach is very different from the attitude in evidence-based, analytical medicine. A patient’s perspective in a problem-solving process can be strongly influenced by diverging and converging ideas and by effectively visualizing a solution into tangible artifacts [6]. For example, identifying patient’s unmet needs for the improvement of person-centered care [7], identifying the causes of problems in the patient engagement process [12], visualizing future services by presenting scenarios and prototypes through workshops [13], or offering strategies by performing persona-scenario sessions [14] could be explained as divergent processes. However, identifying the preferences of patients or clinicians for specific factors [1], analyzing barriers to a particular topic [15], assessing the usefulness of a questionnaire [16], or refining a tool and evaluating the tool’s feasibility [17] correspond to a convergence process.

### 1.2. DT Process

The DT process deals with the overall problem-solving process holistically and integratively by diverging and converging ideas iteratively. This process helps to solve complex and mutually conflicting PE issues flexibly. Applying a patient-centered approach to the problem-solving process is a useful deviation from the system-focused thinking approach.

**Figure 1:**
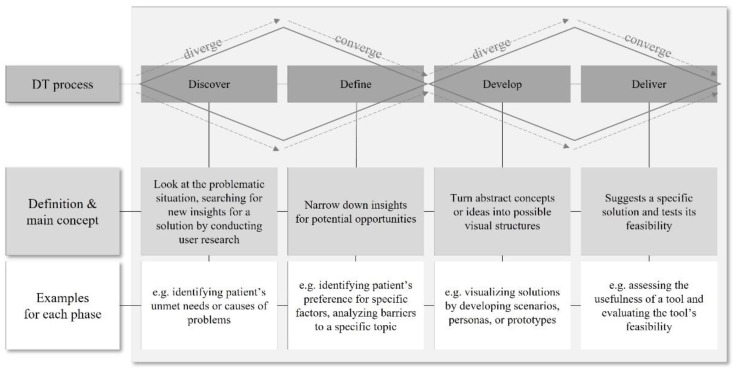
The DT process as a framework for creative problem-solving.

The DT process was originally introduced by the Design Council (2015) [18] and consisted of four stages: “Discover”; “Define”; “Develop”; “Deliver”. Each stage requires collaboration with stakeholders, reflecting multidisciplinary perspectives [19]. “Discover” corresponds to the divergent stage of DT by considering the problematic situation and by searching for new insights to explore potential ideas for a solution by conducting user research. “Define” is the stage of converging the ideas to narrow insights for potential opportunities. “Develop” is the stage of diverging the ideas again to turn abstract concepts or ideas into visual structures as tangible result forms. “Deliver”, as a re-converged stage of ideas, suggests a specific solution to a problematic issue and tests its feasibility (Figure 1).

The DT process can be understood as a framework for viewing problems from an integrated point of view. Therefore, designing the study by including the four stages, rather than focusing on a specific stage in the DT process, can be an effective methodology for PE improvement in healthcare.

### 1.3. DT Themes

DT themes introduced by Carlgren et al. (2016) deal with five important concepts for creative problem-solving: “User focus”, “Problem-framing”, “Visualization”, “Experimentation”, and “Diversity”. They emphasize that creative problem-solving is possible through (1) the deep understanding or involvement of users in identifying their pain points or latent needs by using design ethnography such as user journeys, empathy map, and persona; (2) reframing the initial problem space to catch an unexpected potential solution with the techniques of how-might-we-questions, the five whys technique, and the problem statement; (3) transforming ideas into visual structures to effectively communicate ideas among users by using physical prototypes, sketching, and storyboarding; (4) iterative working for the refinement and testing feasibility of the solution for evaluation by conducting brainstorming; (5) involving multiple teams and the collaborative approach to reflect diverse perspectives in the decision-making process by using study visits, analogies, and demographics [20].

These themes are closely related to the DT process and emphasize holistic, integrative, iterative, and user-centered attitudes by actively engaging users.

Among these themes, “User focus” looked at its contents focusing on user engagement and type. Anderson et al. (2021) classified user engagement approaches for PE improvement into three types according to the degree of intervention. They include (1) “Consultation” by gathering feedback from users through surveys, interviews, and focus groups; (2) “Collaboration” by participating in the discussion, brainstorming, developing solutions, and evaluating results with diverse stakeholders; (3) “Blended approach” that combines “Consultation” and “Collaboration” (co-creation of the solution by working with diverse stakeholders based on the consultation data identified in the early phase) [21]. This classification helps the understanding of how active user engagement was induced to obtain patient-centeredness and how it can guide the design of user engagement methods.

**Figure 2:**
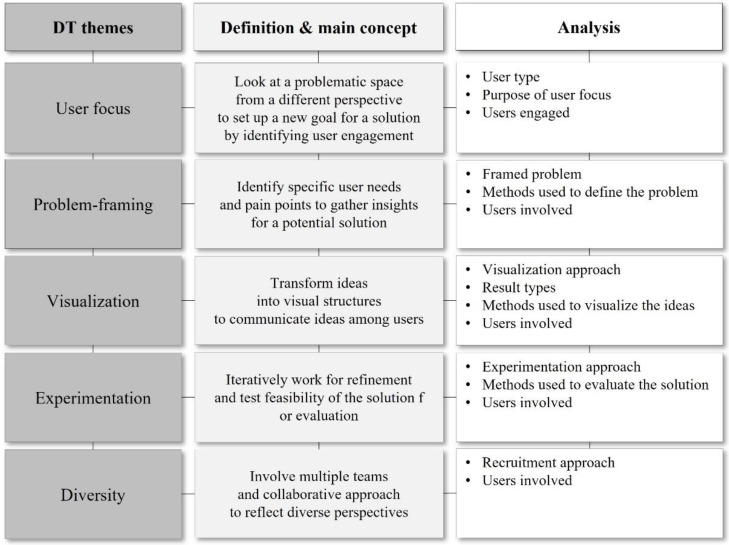
The DT themes representing methodological concepts.

“Problem-framing” involves gathering insights for a potential solution by identifying the specific needs of users. “Visualization” deals with exploring solutions to the problems identified in the previous step by employing divergent thinking and developing them as a tangible prototype. “Experimentation” deals with evaluating and testing the prototypes developed for problem-solving in the last stage by using convergent thinking to focus on the feasibility of the solution. “Diversity” is related to the involvement of diverse members from different backgrounds and the consideration of different user perspectives (Figure 2).

**Figure 3:**
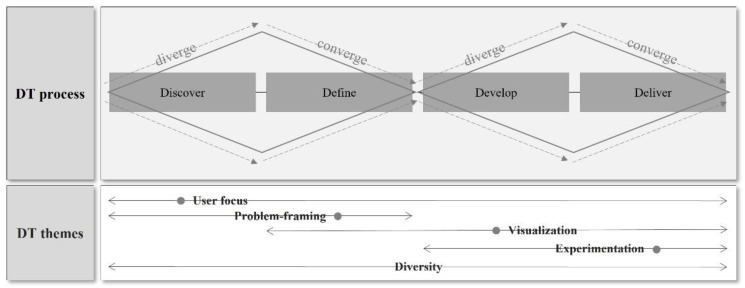
The relationship of DT themes with the DT process.

Figure 3 explains the relationships of five DT themes with the DT process. “User focus” is related to the whole stages of the DT process, mainly focusing on the stage of “Discover”. It helps to set the level of user engagement and decide the type and the scope of participants for employing different perspectives in the overall process. “Problem-framing” is related to the stage of “Discover” and “Define” in the DT process, focusing on the stage of “Define”. It tries to reframe the problem from a new or different perspective and tries to identify user needs. This theme includes divergent and convergent aspects to expand the research scope and narrow down the study purpose to set up a more specific problem-solving goal. In this case, both divergent and convergent thinking must be included, and when only one of them is dealt with, the research scope can become too broad and vague, making it difficult to apply a new perspective; therefore, it cannot be differentiated from the existing traditional problem-solving methods. “Visualization” mainly corresponds to the “Develop” stage, involving “Define” and “Deliver” stages for the iterative work process. It explores the solution in a specific and tangible manner by expanding the search area for possible solutions. “Experimentation” deals with iterative work for the refinement and feasibility test, which corresponds to the “Deliver” stage by narrowing down the solution to a specific result. It also involves the “Develop” stage in the process of complementing the prototype iteratively. “Diversity” involves the whole DT process by affecting the perspectives of users engaged. These five themes are helpful when determining specific methods for each step of the DT process or deciding the comprehensive nature of the research or the characteristics of participants.

The above frameworks, as shown in Figure 1, Figure 2 and Figure 3, suggest essential concepts to understand how holistic and integrative the current studies approached problem situations and how they designed the methods from a patient-centered point of view.

With this background, this study aimed to review how current studies on PEs have approached problem-solving from the DT perspective, and it explored the possibility that the integrative DT approach can be applied as an effective, powerful method to improve the quality of care from the patient perspective.

A scoping review was conducted to examine the approaches and methods used in the current PE-related studies. It was a systematic approach to reviewing the literature on a given topic, providing an overview of the case [22,23,24,25]. To actively achieve patient-centeredness in healthcare, we attempted to draw meaningful insights for applying a holistic, integrative, and practical DT methodology to the medical field. In addition, we aimed to help related researchers increase their understanding of DT to provide insights for dynamically engaging patients in the PE improvement process. The specific research questions were as follows:

RQ 1. What are the characteristics of current studies (study countries, study subjects, and study focus)?
RQ 2. How holistic is the approach and how iterative the process of each study when applied to the DT process?
RQ 3. What approaches and methods were employed, and what user types were involved in the reviewed studies?
RQ 4. How did these studies achieve patient-centeredness in terms of collaboration?

## 2. Materials and Methods

Arksey and O’Malley’s (2005) model [22], a commonly used scoping review method [25], was employed to develop the research framework. It comprises five phases, and the practices described by Tricco et al. (2016) [26] were referred to for each phase. The data were analyzed by detailed coding items corresponding to the research question in each cell using Excel. Based on the method introduced by Levac et al. (2010) [24,27], we performed statistical and thematic analyses based on DT themes and attributes drawn from the literature.

For search strategy, “healthcare”, “QI”, “PE”, “service design” (since service design is more commonly used than DT, it was used in the search condition), “communication”, and “barriers” were used as primary search keywords. For barriers, various terms with similar meanings were used, and terms such as “needs”, “insights”, “protocols”, “evidence”, and “improvements” were additionally used for article search.

**Figure 4:**
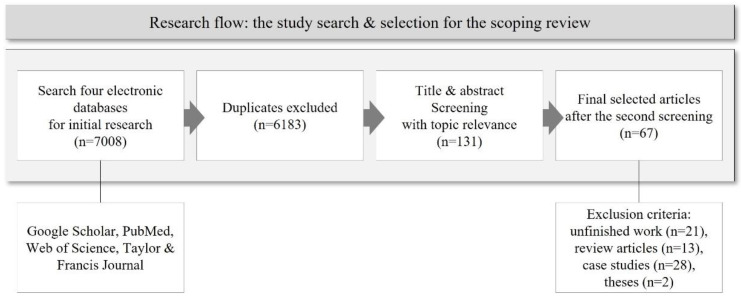
The flow of the research for the scoping review.

Four electronic databases (Google Scholar, PubMed, Web of Science, and Taylor & Francis journal) were used for the study search and selection, and a total of 7008 papers were collected after the primary search. After screening the title and abstract of 6183 papers (excluding duplicates) for relevance to the subject, 131 papers were selected. A total of 67 articles [1,7,12,13,14,15,16,17,21,28,29,30,31,32,33,34,35,36,37,38,39,40,41,42,43,44,45,46,47,48,49,50,51,52,53,54,55,56,57,58,59,60,61,62,63,64,65,66,67,68,69,70,71,72,73,74,75,76,77,78,79,80,81,82,83,84,85] were selected for the final analysis, excluding unfinished work with no results (*n* = 21), review articles (*n* = 13), case studies (*n* = 28), and theses (*n* = 2) (Figure 4).

For the 67 papers selected for the scoping review, study characteristics were examined, focusing on the country where the study was conducted, study subjects, and study focus. The study subjects were classified through a content analysis based on the similarity of the subject range. The study focus was analyzed to determine whether the purpose of the study was to collect user needs or to improve the efficiency of the system or tool.

As the main framework for review, the approaches and methods of the selected studies were examined in two aspects of DT: the process and themes. Each selected study was examined to see how much holistic and iterative work was achieved by identifying which of the four stages of the DT process was applicable. Thereafter, for each of the five DT themes, what specific methods and approaches were applied to each study, what type of participants were involved, and to what extent the DT perspective for creative problem-solving was reflected and used for each analysis were examined.

## 3. Results

### 3.1. Study Characteristics

The distribution of countries where the studies were conducted, by continent, was as follows: 32 studies in North America, showing the highest frequency, followed by 25 studies in Europe. Of the 67 studies, 57 were conducted in North America and Europe, indicating that research to improve PE is the most active in these countries. In Europe, studies for improving PE were conducted mainly in the UK (*n* = 11). Six studies were conducted in Australia and one in Spain in South America. In Africa, two studies were conducted, one in Ghana and one in Nigeria. Joint research between countries was identified in two cases, Denmark and the Netherlands, and 34 countries distributed on various continents. Remarkably, we found no studies based in Asia (Table 1).

From the above results, it is evident that research on PE in healthcare is being intensively conducted in Europe and North America. Contrarily, active research is not being conducted in Africa and Asia, although the importance of a service design for PE is recognized.

The study aims were analyzed in terms of study subjects and study focus. From the content analysis based on the similarity of the subject range covered by each study, the study subjects were classified into five types: data collection and use, user involvement, user needs, care services/intervention, and patient education (Table 2). The issues with the highest frequency were user involvement and user needs (*n* = 24 each), followed by PE data (*n* = 9), care services/intervention (*n* = 8) and patient education (*n* = 2). These results indicate that the recent PE research for QI focused on the unmet needs and involvement of the user-centeredness on the patient rather than on systems such as care service, education, or intervention.

In user involvement, the active engagement (*n* = 14) and communication (*n* = 10) of users were identified as sub-topics, and for user needs, the topics of unmet needs (*n* = 14), user perception, or satisfaction (*n* = 10) were covered. This indicates that the service provider realizes the importance of understanding the users’ needs and direct user participation and interaction in improving the PE. In the data collection and use, the topics of data usage (*n* = 5) and data gathering (*n* = 4) were treated with similar frequencies, indicating that collecting problems and barriers to healthcare experience from the patient’s perspective and processing and managing the collected data for the improvement of PE had the same importance.

The study’s focus was to identify whether the focus of research was on collecting and reflecting users’ opinions and needs or on improving the efficiency of tools or systems. Tool focus includes the improvement of medical decision aids, mobile apps, questionnaires, toolkits, and communication cards. System focus had blood test system, patient engagement hospital plan, primary care system, and outpatient palliative care system. Only two studies focused on both the active understanding of user needs and tool development. A representative case was a study that identified the needs of young asthma patients and developed a mobile app through direct patient participation. Focusing on improving tools and systems was limited to achieving patient-centeredness because its primary purpose was to effectively solve existing structural problems rather than viewing the problematic situation from a whole new point of view for developing a unique solution. As seen in Table 3, several studies still focused on improving tools and systems, and few studies lead to the presentation of new solutions based on profound user needs.

### 3.2. Process for a Holistic and Iterative Approach

The results of examining which stage of the DT process each study included, as presented in Table 4, show that 49 studies dealt with only one stage, followed by two studies that dealt with 14 studies that dealt with two stages and three studies with three stages. No studies were found that dealt with all the stages of the DT process. This clearly shows that the existing research focuses only on a specific stage without iteratively going through divergent and convergent thinking. Since such segmental research design cannot go through the four stages of a flexible thinking process, it is difficult to adopt an approach with an integrated point of view and creative problem-solving.

Among the papers that focused on only one stage of the DT process, four papers corresponded to the “Discover” stage. Rather than an intensive analysis of specific problem situations, these were studies that listened to comprehensive opinions for the improvement of overall care services or systems from the perspective of patients or medical staff. For example, Schäfer et al. (2015) [85] investigated patients’ perceptions of improvement potential in primary care in 34 countries. Rather than limiting the scope to a specific country to find improvement points, it provided insights to compare differences in the patients’ perspectives by country and set a differentiated improvement direction for each country in the future.

Most of the papers (*n* = 31) were found in the “Define” stage. They focused on discovering pain points for immediate improvement by identifying users’ preferences or needs for a series of interventions or initiatives in detail. For example, Song et al. (2020) [83] identified specific barriers when implementing PE surveys and discussed using their findings for service improvement. This was to find ways to improve and utilize a particular survey tool. It corresponds to a deep dive into a problem space to specifically solve the previously identified problem situation rather than discovering the problem from a new perspective.

There are two papers in the “Develop” stage. Revenäs et al. (2015) [13] developed scenarios and prototypes through a co-design workshop to improve the system requirements and specifications for the mobile Internet service. This study transformed the solution for a specific problem situation into a visible form, enabling it to present results at a more realistic level.

There were 12 studies corresponding to “Deliver”. Lyes et al. (2020) [39] evaluated the extent to which the questionnaire for assessing patient satisfaction in the intensive care unit reflects the patient’s opinion. Miatello et al. (2018) [50] examined the effectiveness of smartphone and web apps for PE data collection. This is to evaluate how effective a particular tool is, and it is necessary for the continuous development and update of the device or system.

In studies involving only one stage of the DT process, the convergent thinking stage of “Define” and “Deliver” appeared the most. This has limitations in creative problem-solving because it is difficult to expand the problem space allowing for flexible thinking due to the absence of divergent thinking stages. Each stage of the DT process has integrity and value as substantial research. However, each stage has different functions and objectives. It is possible to present more detailed and in-depth results when research is designed with continuity rather than proceeding in an independent form. The holistic approach of (1) raising a problem from a new perspective (Discover), (2) analyzing the cause of a specific problem (Define), (3) approaching a possible solution visually and structurally (Develop), and (4) evaluating its usefulness and developing it into a more successful result (Deliver) corresponds to a creative research process that recognizes and solves more real problem situations in patient-centered communication.

In studies involving two stages, 14 cases appeared in the “Define” and “Develop” stages. Since this is an approach of searching for solutions based on problems that have been directly exposed, it is difficult to understand the patient’s latent needs. Moreover, it is hard to reflect a patient-centered perspective in the problem-solving process because the healthcare experts usually hold strong opinions. For example, Fitch et al. (2021) [49] reviewed the main challenges of adolescent and young adult cancer survivors and discussed their suggestions regarding care improvements. However, the needs data collected from patients were not actively utilized. They did not lead to a practical solution because a specific prototype was not developed, and the delivery stage was not progressed to evaluate its usefulness.

Research involving three stages showed integrative thinking and iterative problem-solving processes, but the “Discover” stage was still missing. For example, identifying barriers to the current healthcare system helps researchers focus on the specific problematic situation, but understanding the system’s mechanism from a patient’s perspective can help healthcare researchers set up a new goal for problem-solving.

As such, studies in which the stages of the DT process are not continuously applied hold significant values in themselves. However, from a macro perspective, to creatively solve complex and difficult-to-solve problems in the medical field by adding a different and new perspective, it is necessary to apply each DT stage responsible for other functions continuously.

### 3.3. DT Themes for a User-Centered Approach

#### 3.3.1. User Focus Aspect

Concerning the user types who participated in each study, 23 studies included patients and/or families, and 18 showed the highest frequency of patient participation compared to other users. The involvement of medical staff and/or caregivers and professionals was identified in 12 studies, and the participation of professionals, healthcare leaders, and society members was identified in four cases. Patients and medical staff participated together in 26 cases, which accounted for the most significant proportion among user types, and the mixed participation of patients and professionals was reported in two cases (Table 5).

When examining the significance of user participation, user involvement was the most frequent topic, with 34 cases, followed by user understanding, with 26 cases. Thus, it is evident that, for PE improvement, users’ direct participation is crucial to understand their needs rather than just contacting users. Seven studies combined user understanding and participation, indicating a significant attempt to involve users in deriving improvement directions directly and understanding user needs to improve the PE (Table 6).

However, it is interesting to note that 48 cases of consultation, 12 cases of a blended approach, and seven cases of collaboration were reported in user engagement. This implies that the users’ participation activity is more of a passive and one-way opinion gathering rather than an active reflection of the users’ opinion and interaction with other stakeholders. When users include patients, user engagement at the consultation level is higher. These results suggest that researchers recognize the importance of direct patient participation and are trying to engage them for patient-centered care. However, they are still accustomed to methods focused on simple data collection from the users rather than managing active interaction.

#### 3.3.2. Problem-Framing Aspect

For the problems framed at this stage, identifying users’ needs/barriers/preferences/satisfaction had the highest number of cases, with 33 cases, followed by understanding the current tool or system, with 19 cases. The most common methods used for ‘Problem- framing’ were interviews (*n* = 34), surveys (*n* = 21), and focus groups (*n* = 11). The focus was on identifying insights on specific issues directly from stakeholders, which means that the current studies lack the efforts to reframe the initial problem by reflecting the viewpoints of various users. It is difficult to achieve a creative problem-solving approach when the focus is on identifying the user’s needs within the range of a problem that has already been established rather than reframing the initial problem.

In addition, for the framed problems, while identifying users’ needs and analyzing issues with existing systems and tools that were mainly used, access to intangible problems such as users’ emotions, attitudes, and perceptions was relatively lacking. However, since these factors are critical in understanding the patient’s potential needs, a balance of research to understand the emotions and attitudes of patients is needed, along with simple satisfaction or preference-based needs analysis. As for the method of defining the problem, the questionnaire was frequently used following the interview, clearly showing that there is a limit to understanding the patient’s inner voice. The scope of users related to the problem-framing aspect involved mainly direct stakeholders such as patients, medical staff, and caregivers. To understand the patient’s unmet needs, the participation of experts in different fields such as cognition and psychology can help for an in-depth problem-framing approach (Table 7).

#### 3.3.3. Visualization Aspect

As for the “Visualization” approach, visualization of suggestions/strategies accounted for the most with seven cases (e.g., creating personas and scenarios to suggest strategies or building a visual structure of suggestions), followed by storyboarding ideas for tool/system development (e.g., developing a film to share and collect feedback from users) with four cases and mapping needs for priority setting with four cases. In three cases, the detailed specifications of a prototype (e.g., refining the existing decision aid tool) and the development of new prototypes (e.g., a mobile app or screening tool as a checklist) were found in three cases. This approach has been introduced in various formats. However, most of them have remained at the level of suggesting conceptual ideas for improvement rather than developing a tangible prototype, thus failing to actively explore the opportunity of creating a solution.

Most result types (*n* = 18) corresponded to visible software such as a mobile app, tool/program, film, or more abstract suggestions, strategies, processes, models, and interventions. Only one whiteboard design was identified as the hardware. The results were verified only in five studies, and a user test, focus group, audit, and interview were used for prototype evaluation. These results showed that an abstract conceptual discussion prevails over tangible results in this process.

As a visualization method, it was found that workshops involving various stakeholders were the most used. The active participation of users and the reflection of opinions were achieved to some extent in the visualization stage.

Most of the users involved in the visualization method were direct stakeholders such as patients, medical staff, and caregivers, but what stands out is that some experts, such as the design team, were included in the visualization process. It was interpreted that the designer’s role is necessary for developing a high-fidelity prototype. The number of designer-participating cases was still as few as four, indicating that workshops made up of professionals in various fields are needed for the active exploration of tangible prototypes as solutions to healthcare problems (Table 8).

#### 3.3.4. Experimentation Aspect

For the “Experimentation” approach, five approaches were identified: (1) providing feedback for improvement (*n* = 4); (2) testing the feasibility of the tool/program (*n* = 5); (3) discussing the prototypes for improvement (*n* = 5); (4) evaluating the values of the prototypes (*n* = 5); and (5) prioritizing the recommendations (*n* = 1). A survey, focus group, interview, and trial test were used as methods, and users involved in the “Experimentation” showed various configurations (Table 9).

The “Experimentation” aspect is related to verifying feasibility through testing different solutions, and various users must participate and work iteratively to explore the answers. However, since many studies use questionnaires to receive user feedback, there is a limit to interrelated collaborative work between users by employing personal meetings or surveys. For users to actively present their opinions, test various solutions, and converge their thoughts to a specific result, it is necessary to apply an interactive method for brainstorming, such as a workshop.

#### 3.3.5. Diversity Aspect

Regarding the “Diversity” aspect of DT themes, the recruitment approach was classified as internal/external to identify team composition and diversity of viewpoints. For 52 out of the 67 studies, there was external recruitment of the team members, and for studies focused on specific healthcare settings, team members were recruited internally (*n* = 15).

User involvement was analyzed by categorizing the user groups into direct and indirect stakeholders. Of the selected studies, 51 involved only direct stakeholders, showing the highest frequency. A mixture of direct and indirect stakeholders was reported in eight studies, and four studies reflected diverse viewpoints by including non-medical staff and professionals such as a design team, a game researcher, a program development team, and a multidisciplinary team.

Nineteen studies directly mentioned EBCD, co-design, and participatory design as methods to reflect the perspectives of various users. Most of these researchers (*n* = 14) preferred involving direct stakeholders obtained through external recruitment (*n* = 13). The results showed that the EBCD studies were active in acquiring diversity in recruitment but were limited to specific users and viewpoints in applying user involvement and DT themes. Although collaboration with external factors has been sufficiently achieved in terms of the recruitment approach, it is insufficient to reflect a multidisciplinary perspective on user participation types (Table 10).

## 4. Discussion

The following insights were derived when examining the approaches and methods of the research for PE improvement.

First, in the distribution of countries, most of the studies centered on North America and Europe, while South America, Africa, and Asia hardly appeared. Considering that the overall quality of life in countries has improved and the importance of PE and the level of patient expectations have increased, active research should also be introduced in these countries.

Second, the user’s perspective was treated as necessary in the study subject, focusing on topics related to user participation, interaction, needs, and satisfaction. However, patient feedback, data utilization, patient education, and intervention showed relatively low proportions; therefore, it is necessary to expand the scope to more diverse research topics in the future.

Third, when selected studies were viewed in the DT process, rather than dealing with the iterative process of divergent and convergent thinking, the focus was on the stages of “Define” and “Deliver”, which correspond to the convergent thinking for problem identification. Given that medicine is accustomed to the analytical approach corresponding to convergent thinking, a holistic approach through the iteration of divergent and convergent thinking should be introduced to enable flexible and creative problem-solving.

Fourth, in terms of the “User focus” aspect, which corresponds to understanding user needs and using qualitative user research methods through empathy, user involvement at the consult level was higher. It indicated that user participation was increased, but it was still at the level of simple one-way communication. As user participation is still at the level of passive and one-way opinion listening, it is necessary to deeply understand and empathize with the patient’s needs by applying ethnographic design techniques, such as “persona”, “scenario”, and “user journey”. This approach allows researchers to solve problems with the question of “why” rather than “what” by observing, experiencing, and interviewing users from a patient’s perspective.

Fifth, in terms of the “Problem-framing” aspect relevant to reframing the problem space from a user perspective, the current study’s scope was confined to the stage of “Define”. It mainly focused on presenting the current problems rather than discovering the potential needs of patients and redefining previously unseen problems. For creative problem-solving to improve PE, it is essential to share and interpret problems discovered through site visits and user research with various members and reframe the problem to find potential opportunities. To define the problem from the user’s perspective by understanding the behavior and emotions of the patient through “Persona”, a strategic fictional person based on precise observation information and “Patient journey map”, a user-centered analysis tool, can be used. Therefore, problems related to PE in healthcare require a unique perspective on the problem and restructuring based on user research from the patient’s point of view.

Sixth, for “Visualization”, which explores creative solutions by co-creating with various users through divergent thinking, the current studies showed relatively low weight compared to other aspects. “Visualization” provides a tangible communication platform through visual prototyping. Thus, it is helpful for users to exchange opinions more efficiently and to materialize solutions by using the specified artifacts. To obtain a tangible result, relevant experts must participate in each stage of the research and be able to communicate and share their opinions while presenting solutions. Therefore, this aspect, which enables divergent thinking for creative solutions through the participation and interaction of various users, should be actively employed as a methodology for improving the patient-centered healthcare experience. For this, active communication through multidisciplinary team formation and co-creative workshops are required to develop ideas into specific and tangible prototypes.

Seventh, “Experimentation” is a process of iteratively exploring feasibility through testing and verifying solutions that have been explored in the “Visualization” stage. This aspect presents practical and tangible results by narrowing the scope of the divergent thinking back to a concrete solution. However, it is challenging to derive various and creative solutions from current studies because they directly extract keys from the problems identified analytically in the “Define” stage while skipping the “Develop” stage, which explores diverse possibilities. Thus, for developing creative solutions to achieve patient-centeredness, the aspect of “Experimentation” relevant to the attributes of iterative, collaborative, and convergent attributes in DT should be employed. It would be more effective as a methodology for creative problem-solving when various users incorporate methods such as brainstorming.

Lastly, in terms of “Diversity” relevant to the various configurations and viewpoints of the participating users, the recruitment approach is still limited to a specific stakeholder group, thereby not fully reflecting a multidisciplinary perspective in terms of involving diverse professionals from a variety of fields. To fulfill the “Diversity” aspect, it is necessary to form a team with various users and stakeholders for the PE improvement by consciously recruiting team members from a variety of fields such as doctors, UX designers, programmers, and psychologists and by conducting broad research to enable open communication among participants. In this process, a holistic perspective and integrative thinking can be developed, which suggest meaningful insights for improving patient-centered experiences in healthcare.

## 5. Conclusions

This study systematically viewed current studies on PE in healthcare from the DT perspective to explore the future applicability of the DT methodology that can apply an integrative approach and derive creative solutions through the iterative process of divergent and convergent thinking. The viewpoint of DT was reviewed in this study by employing the four-staged DT process and the five DT themes. As a result, it was found that the DT process is helpful as an approach for designing or evaluating a holistic and iterative research process. Dt themes presented criteria for designing and evaluating specific methodologies for each process step. However, the DT process and themes presented in this study provide a conceptual framework as a new methodology for creative problem-solving in the future medical field. Therefore, for this new approach to be actually applied to healthcare practice, application cases and the evaluation of specific methods should be made.

A patient-centered approach is vital for improving PE. For this purpose, it is meaningful to apply the DT methodology that emphasizes the holistic approach and iterative process of diverging and converging an idea to a problem space for healthcare innovation. This study has the potential to provide significant insights for healthcare researchers to seek a practical methodology for patient-centered healthcare improvement by introducing the concept of DT.

## Figures and Tables

**Table 1:** Summary of the study countries and continents.

Continent	Countries	Count	Total (%)
Africa	Ghana	1 [15]	2 (2.99%)
	Nigeria	1 [28]	
Europe	Belgium	1 [29]	25 (37.31%)
	Denmark	1 [30]	
	France	1 [16]	
	Germany	1 [17]	
	Netherlands	3 [12,31,32]	
	Norway	1 [7]	
	Sweden	4 [13,33,34,35]	
	Switzerland	1 [36]	
	UK	11 [37,38,39,40,41,42,43,44,45,46,47]	
	Denmark/Netherlands	1 [48]	
North America	Canada	8 [14,21,49,50,51,52,53,54]	32 (47.76%)
	USA	24 [55,56,57,58,59,60,61,62,63,64,65,66,67,68,69,70,71,72,73,74,75,76,77,78]	
South America	Spain	1 [79]	1 (1.49%)
Australia	Australia	6 [1,80,81,82,83,84]	6 (8.96%)
Multiple continents	34 countries	1 [85]	1 (1.49%)
Total	-	67	67 (100%)

**Table 2:** Summary of study subjects.

Study Subjects			Count (%)
Focusing on patient data collection and use	The use of PE data	5	9 (13.43%)
	Capturing feedback/gathering data from patients	4	
User involvement	User engagement for QI	14	24 (35.82%)
	Reciprocity/communication among users	10	
Identifying user needs	User perception/satisfaction to care	10	24 (35.82%)
	Unmet needs among users	14	
Care services/intervention	Care services to be improved	5	8 (11.94%)
	Intervention for improvement	3	
Patient education	Patient education	2	2 (2.99%)
Total		67	67 (100%)

**Table 3:** Summary of study focus.

Study Focus	User-Focused	Tool-Focused	System-Focused	User/Tool-Focused
Count (%)	32 (47.76%) [1,7,15,28,30,31,32,33,34,35,37,40,43,45,47,48,49,51,52,56,65,67,68,71,72,73,74,78,79,80,82,84]	21 (31.34%) [12,13,14,16,17,29,36,39,44,46,50,53,55,57,58,59,62,66,70,75,77]	12 (17.91%) [21,38,41,42,54,60,61,63,64,76,83,85]	2 (2.99%) [69,81]
Total	67 (100%)			

**Table 4:** Summary of the study stage in the DT process.

DT Stage	Discover	Define	Develop	Deliver	Count (%)
Involve one stage	[7,12,41,85]				4 (5.97%)
		[1,15,28,29,30,31,32,33,34,40,42,45,48,54,60,65,66,67,68,70,71,72,73,74,75,76,78,79,82,83,84]			31 (46.27%)
			[13,14]		2 (2.99%)
				[16,17,36,39,46,50,53,55,58,61,62,63]	12 (17.91%)
Involve two stages		[21,35,37,43,44,49,51,52,56,57,64,69,80,81]			14 (20.89%)
Involve three stages		[38,47,77]			3 (4.48%)
Total					67 (100%)

**Table 5:** Summary of user types.

User Types		Count (%)	
Involving patients/families	Patients	18	23 (34.33%)
	Patients/caregivers	4	
	Caregivers (families)	1	
Involving medical staff	Medical staff	7	12 (17.91%)
	Medical staff/caregivers	2	
	Medical staff/professionals/researchers	3	
Involving professionals	Professionals/leaders/society members	4	4 (5.97%)
Involving patients/medical staff	Patients/medical staff	12	26 (38.81%)
	Patients/caregivers/medical staff	8	
	Patients/medical staff/professionals	4	
	Patients/medical staff/development team (designers)	2	
Involving Patients/professionals	Patients/professionals	1	2 (2.98%)
	Patients/caregivers/professionals	1	
Total	-		67 (100%)

**Table 6:** Purpose of user focus and engagement aspects.

Category	Result		Count (%)	
Purpose of user focus	User understanding	[1,7,15,28,30,31,32,33,34,35,40,45,48,49,65,67,68,69,71,72,73,74,78,79,82,84]	26 (38.80%)	67 (100%)
	User involvement	[12,13,14,16,17,21,29,36,38,39,41,42,44,46,50,51,53,54,55,57,58,59,60,61,62,63,64,66,70,75,76,77,83,85]	34 (50.75%)	
	User understanding and involvement	[37,43,47,52,56,80,81]	7 (10.45%)	
User engagement	Consultation	[1,12,15,16,17,21,29,30,31,32,33,34,35,40,41,45,46,48,49,50,53,54,55,56,58,60,61,62,63,64,65,66,67,68,69,70,71,72,73,74,75,76,78,79,82,83,84,85]	48 (71.64%)	67 (100%)
	Collaboration	[7,13,14,28,36,42,59]	7 (10.45%)	
	Blended (co-creation)	[37,38,39,43,44,47,51,52,57,77,80,81]	12 (17.91%)	

**Table 7:** Summary of study’s problem-framing.

Perspective	Description	Count (*n*)	
Framed problem	Users’ needs/barriers/pain points/preferences/satisfactions	33	62
	Current/existing tool or system	19	
	Users’ perception/knowledge/attitudes/emotions	6	
	Potential improvement of the current system	4	
Methods used to define the problem	Interviews	34	73
	Focus group	11	
	Workshop	4	
	Survey (Delphi)	21	
	Checklist	1	
	Observation	2	
Users involved	Patients	21	58
	Patients/caregivers	4	
	Caregivers	3	
	Patients/medical staff	10	
	Patients/caregivers/medical staff	3	
	Medical staff	9	
	Professionals	2	
	Medical staff/professionals	6	
	Patients/caregivers/medical staff/professionals	3	

**Table 8:** Summary of the study’s visualization.

Perspective		Description	Count (*n*)	
Visualization approach		Visualization of suggestions/strategies	7	21
		Storyboarding ideas for tool/system development	4	
		Mapping the needs for priority setting	4	
		Provide/develop detailed specifications	3	
		Develop prototypes	3	
Result types	Software	Mobile app	4	19
		Visual structure of suggestions/strategies	7	
		Film	1	
		Tool/program	4	
		Process/model	1	
		Intervention	1	
	Hardware	Whiteboard	1	1
Methods used to visualize the ideas		Interviews	4	22
		Focus group	6	
		Workshop	10	
		Survey	2	
Users involved		Patients	3	21
		Patients/caregivers	1	
		Patients/medical staff	1	
		Patients/professionals	1	
		Patients/caregivers/medical staff	5	
		Patients/medical staff/professionals	1	
		Medical staff	3	
		Professionals (design team)	4	
		Medical staff/professionals	1	

**Table 9:** Summary of the study’s experimentation.

Perspective	Description	Count (*n*)	
Experimentation approach	Provide feedback for improvement of the existing tool	4	20
	Test the feasibility of the program/tool	5	
	Discuss the prototypes for improvement	5	
	Evaluate the values of the results (prototypes)	5	
	Rate and prioritize the recommendations	1	
Method used to evaluate the solution	In-person meeting	1	26
	Focus group	5	
	Feedback session	1	
	Survey (Delphi)	8	
	Checklist	1	
	Audit	1	
	Interview	5	
	Workshop	1	
	Prototype test as a trial	3	
Users involved	Patients	5	19
	Patients/caregivers/medical staff	3	
	Patients/medical staff	2	
	Patients/professionals	1	
	Patients/medical staff/professionals	2	
	Medical staff	2	
	Medical staff/caregivers	2	
	Caregivers	1	
	Professionals	1	

**Table 10:** Summary of the study’s diversity aspects.

Perspective		Description	General Count		EBCD Count
Recruitment approach		Internal recruitment	15		6
		External recruitment	52		13
Users Involved	Direct stakeholders	Patients	20	51	14
		Patients/caregivers/medical staff	8		
		Patients/medical staff	10		
		Patients/caregivers	3		
		Caregivers/medical staff	2		
		Medical staff	7		
		Caregivers	1		
	Indirect stakeholders	Interprofessional team (non-clinical team)	1	4	1
		Professionals	3		
	Mixed stakeholders	Patients/caregivers/medical staff/professionals	2	8	1
		Medical staff/professionals	4		
		Patients/medical staff/professionals	2		
	Multidisciplinary perspectives	Multidisciplinary team (adolescents, health informaticians, medical anthropologist, psychiatrist, gaming and digital media researcher)	1	4	3
		Patients/medical staff/design team	1		
		Patients/professionals (digital game researcher)	1		
		Patients/medical staff/program development team	1		
